# Distinct non-clock-like signatures of the basal cell carcinomas from three sisters with a lethal Gorlin-Goltz syndrome

**DOI:** 10.1186/s12920-022-01324-7

**Published:** 2022-08-05

**Authors:** Lihua Ye, Li Wang, Kexin Peng, Ou Fang, Zhen Tian, Caihua Li, Xiaopeng Fu, Qingdong Chen, Jia Chen, Jing Luan, Zhenghua Zhang, Qiaoan Zhang

**Affiliations:** 1 Department of Dermatology, Haikou People’s Hospital, Xiangya Medical College, Central South University, Hainan, China; 2 Department of Dermatology, Huashan Hospital, Shanghai Medical College, Fudan University, Shanghai, China; 3 Genesky Biotechnologies Inc, Shanghai, China; 4 Department of Dermatology, Dongfang People’s Hospital, Hainan, China; 5 Department of Dermatopathology, Shanghai Skin Disease Hospital, School of Medicine, Tongji University, Shanghai, China

**Keywords:** Gorlin-Goltz syndrome, PTCH1, Mutational signatures

## Abstract

**Background:**

Gorlin*-*Goltz syndrome (GS) is an inherited disease characterized by predisposition to basal cell carcinomas (BCCs) and various developmental defects, whose numerous disease-causing *PTCH1* mutations have been identified in the hedgehog (Hh) signaling pathway.

**Methods:**

In this study, whole exome sequencing was used to screen for both somatic and germline deleterious mutations in three sisters with a lethal GS. The mutations we found were confirmed by subcloning and Sanger sequencing of the genomic DNA. RNA-seq was performed to profile gene expression in paired BCCs samples and the expression levels for selected genes were validated by quantitative PCR.

**Results:**

The clinical and histopathologic features were analyzed for the proband in the three-generation GS family. We identified the insertion mutation *PTCH1* c.1341dupA (p. L448Tfs*49), which segregated with BCC phenotype and contributed to the death of two in four patients from a Chinese family with GS. Compared with adjacent non-cancerous tissues (ANCT), four second-hit mutations were found in four of the six pairs of BCC from three patients. Of note, somatic genomic alterations in all six BCC samples were mainly clustered into non-clock-like Signature 7 (ultraviolet mutagenesis) and 11 (related to certain alkylating agents). Both RNA-seq and quantitative RT-PCR confirmed that the mRNA levels of *PTCH1* and its effector *GLI1* were markedly upregulated in six pairs of BCC samples versus ANCT.

**Conclusions:**

The distinct non-clock-like signatures of BCCs indicated that GS was not a life-threatening illness. The main reasons for untimely death of GS patients were *PTCH1* mutation, exposure to intense ultraviolet radiationand the poor economic conditions.

**Supplementary Information:**

The online version contains supplementary material available at 10.1186/s12920-022-01324-7.

## Background

Gorlin-Goltz syndrome (GS, OMIM #109400), also called nevoid basal cell carcinoma syndrome (NBCCS), is a rare autosomal dominant disorder that predispose to early onset tumours, such as multiple BCCs and medulloblastoma [1]. It is generally recognized that the life expectancy is not significantly affected by GS [2]. Here, we collected one three-generation family with GS from Hainan province of China. Unfortunately, two patients died of cachexia resulting from aggressive BCCs at the age of 54 and 39 years, respectively. To investigate the genetic cause of the untimely death, we performed WES to detect the causative germline and somatic mutations. Moreover, RNA-Seq based transcriptomics, combined with quantitative RT-PCR, were used to determine preferential expressions of the genes related to GS. It is of note that GS is mostly caused by mutations in the patched 1 (*PTCH1*) gene in hedgehog (Hh) signaling pathway [3]. More recently, patched 2 (*PTCH2*) is no longer considered a highly susceptible gene for GS [4]. Although the germline mutation in *PTCH1* c.1341dupA (p. L448Tfs*49) we found in this family has been listed in the Human Gene Mutation Database (HGMD^®^), no clinical data are available from those patients. This study provided the clinical information and *PTCH1* mutations identified in three sisters with a lethal GS.

## Methods

### Sample collection

**Fig. 1:**
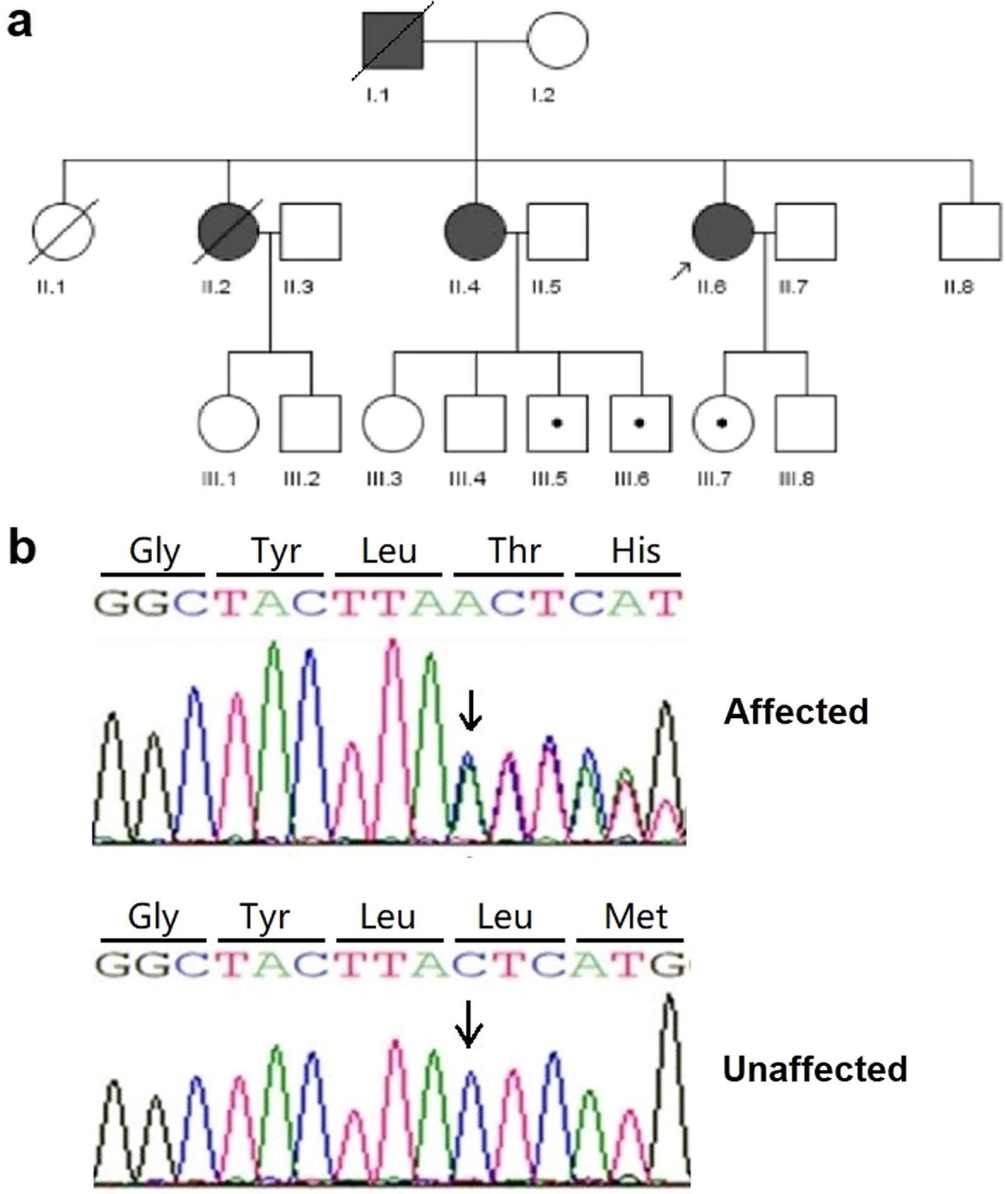
**a** The pedigree chart of three-generation familial GS. Squares and circles denote males and females, respectively. Affected and unaffected individuals are represented by black and open symbols, respectively. Slash lines and dot marks indicate death and disease-carrying genotype, respectively. The initial proband was indicated by an arrow. **b** Sanger sequencing chromatograms of proband (II-6, affected) and normal control (II-7, unaffected) at the c.1341dupA mutation site indicated by arrow

A three-generation GS family was identified from Hainan Province of China (Fig. 1a). All family members and non-familial cases were carefully examined by at least 2 dermatologists, and the diagnosis was confirmed by histological examination of skin biopsy specimens on the proband (II-6). With patents’ written informed consent, 13 blood samples were collected from the family members. Moreover, six pairwise BCCs and ANCT were dissected from 3 affected females (II-2, II-4, II-6). All procedures followed the guidelines of the Helsinki Declaration and were approved by the Scientific Ethics Committee of Affiliated Haikou People’s Hospital, Xiangya School of Medicine.

### Whole exome sequencing (WES) and data analyses

Genomic DNA (gDNA) was extracted from peripheral blood and the frozen tissues with DNeasy Blood & Tissue Kit (Qiagen, Germany) according to the manufacturer’s protocol. The purity and concentration of the gDNA met the sequencing requirements. WES was performed using gDNA with a SureSelectXT Reagent kit (Agilent, USA), SureSelectXT Human All Exon V6 (Agilent, USA), TruSeq PE Cluster Kit v3-cBot-HS (Illumina, USA), and HiSeq SBS Kit V4 (Illumina, USA). Quantification was performed with an Agilent Bioanalyzer (Agilent Technologies, USA), and multiplexed sequencing was done on HiSeq 2500 sequencers with 2 × 150 paired-end modules (Illumina, USA). Total sequencing depth was performed at 100× coverage.

Before variant calling, the raw sequence reads were mapped to human genome reference (hg19). Then, PCR duplicates were marked to mitigate the possible biases and the base quality scores were recalibrated by using the Genome Analysis Toolkit (GATK) [5]. For germline single nucleotide variants (SNVs) and insertion/deletion variants (indels), the GATK4 HaplotypeCaller was used to produce a genomic variant call format (GVCF) file. Next, the GVCFs from multiple samples were consolidated into a GenomicsDB datastore, followed by joint genotyping and variant quality score recalibration (VQSR) filtering to produce the final callsets with the balance between sensitivity and precision [5]. Exome-wide sequence variants were filtered with the sequencing depth < 5×. For somatic short variants (SNVs and Indels), the GATK4 MuTect2 was used to analyze somatic mutations in BCCs and ANCT, and the confident somatic calls were filtered by GATK4 FilterMutectCalls and FilterByOrientationBias tools [5].

The Ensembl variant effect predictor (VEP) and vcf2maf tools were applied to generate the somatic mutation annotation format (MAF) files [6]. Moreover, ANNOVAR was also used to annotate population frequencies of variations [7]. The variants were identified as low-frequent functional mutation with < 0.01 frequency in ExAC03 database [8] and 1000 genome database [9], < 0.05 frequency in GeneskyDB database. According to the results, the variants were extracted as one of these functional annotations: “Frame_Shift_Del”, “Frame_Shift_Ins”, “In_Frame_Del”, “In_Frame_Ins”, “Missense_Mutation”, “Nonsense_Mutation”, “Nonstop_Mutation”, “Splice_Site” or “Translation_Start_Site”.

### Subcloning and Sanger re-sequencing of the genomic DNA

Genomic DNA was extracted from each pair of BCCs and ANCT using a TIANamp Genomic DNA Kit (TransGen Biotech, Beijing, China). PCR amplification products were subcloned into the pEASY^®^-Blunt Cloning Vector (TransGen Biotech, Beijing, China) followed by Escherichia coli transformation and blue-white screening. The colonies were analyzed by colony PCR, moreover, capillary electrophoresis was performed on an ABI Prism 3130xl Genetic Analyzer (Applied Biosystems, USA) to screen the somatic mutations through the diverse length of colony PCR products. Fragment analysis was performed using the Peak Scanner Software v1.0 (Applied Biosystems, USA).

### RNA-seq

RNA quality was accessed by an Agilent Bioanalyzer and those samples with RNA integrity number (RIN) higher than 7 were used to construct RNA-seq libraries by adapter ligation. RNA-seq libraries were prepared from 1 μg of total RNA using VAHTS mRNA-seq V3 Library Prep Kit (Vazyme Biotech) according to manufacturer’s instruction. mRNA was purified with oligo-dT magnetic beads, followed by fragmentation, end repair, adapter ligation and PCR amplification. The analysis of final RNA quality and integrity was performed with Agilent BioAnalyzer and Qubit Fluorometer (Invitrogen, USA). All libraries were sequenced on Illumina NovaSeq platform to generate 150 bp paired-end reads. Raw sequencing data (.fastq files) was processed using STAR (spliced transcripts alignment to a reference) to generate read alignments with hg19. Effective read counts for genes were obtained with featureCounts under default settings, standardized and analyzed using DEseq2.

### Real-time quantitative PCR (qPCR)

The mRNA expression levels of *PTCH1* (NM_000264.3) and *GLI1* (NM_005269.3) were evaluated by real-time qPCR, using total RNA from BCC and ANCT from three sisters with GS. The total RNA of each sample was reverse transcribed to cDNA using the PrimeScript RT Reagent Kit (Takra, Japan). qPCR reactions were conducted in triplicate using the TB Green Premix Ex Taq II (Takara, Japan) and the ABI 7300 system (Applied Biosystems, USA). The primers were designed with the Primer 3 software (http://bioinfo.ut.ee/primer3-0.4.0). qRT-PCR data were analyzed by two-tailed paired-sample t-tests and statistical comparisons were considered significantly different at P < 0.01.

## Results

### Clinical features

**Fig. 2:**
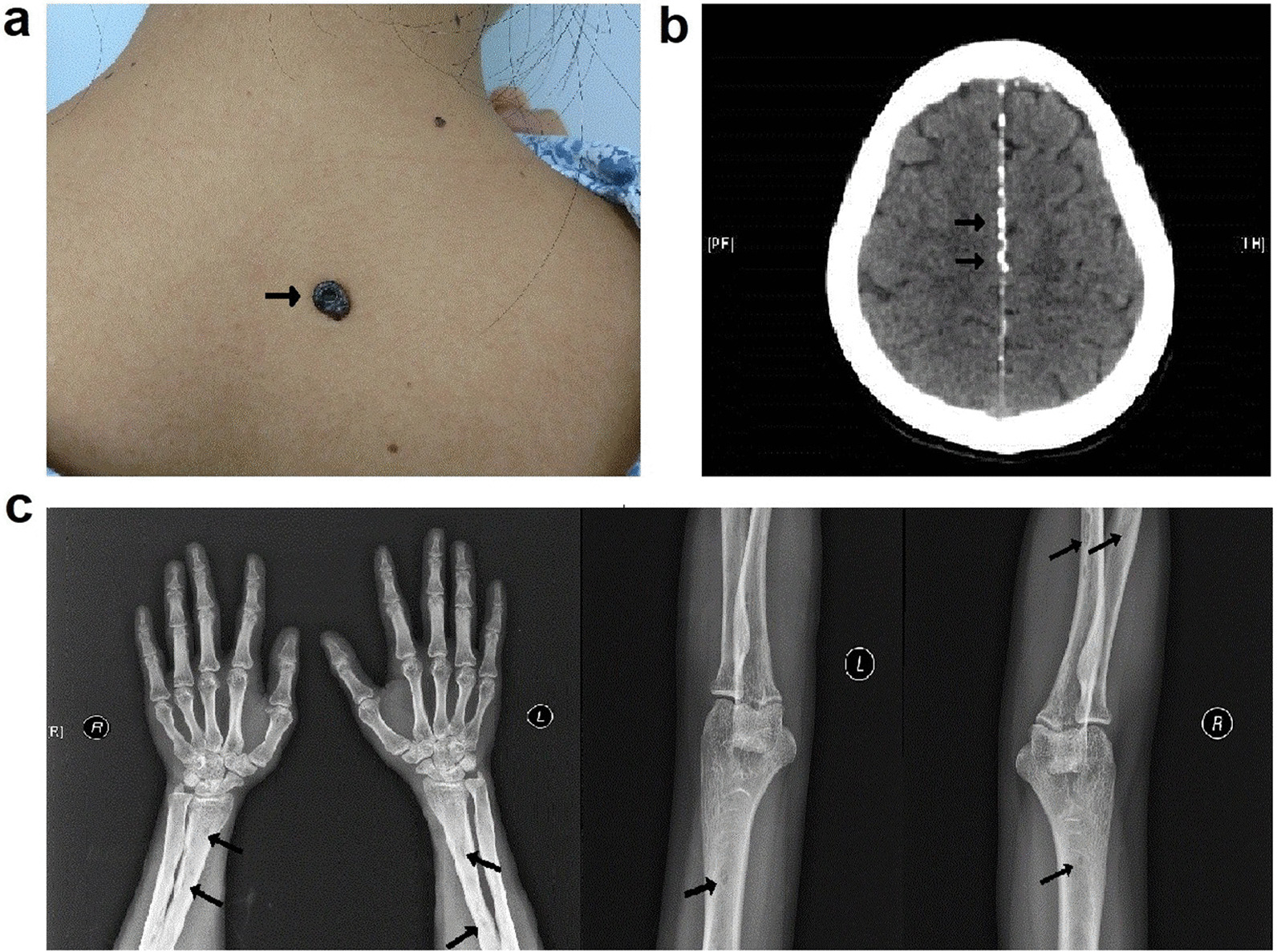
BCC lesions on the back (**a**), falx cerebri calcification on cranial CT **b** and low-density shadow on upper-limb X-ray **c** of the proband, which is indicated by arrows

**Fig. 3:**
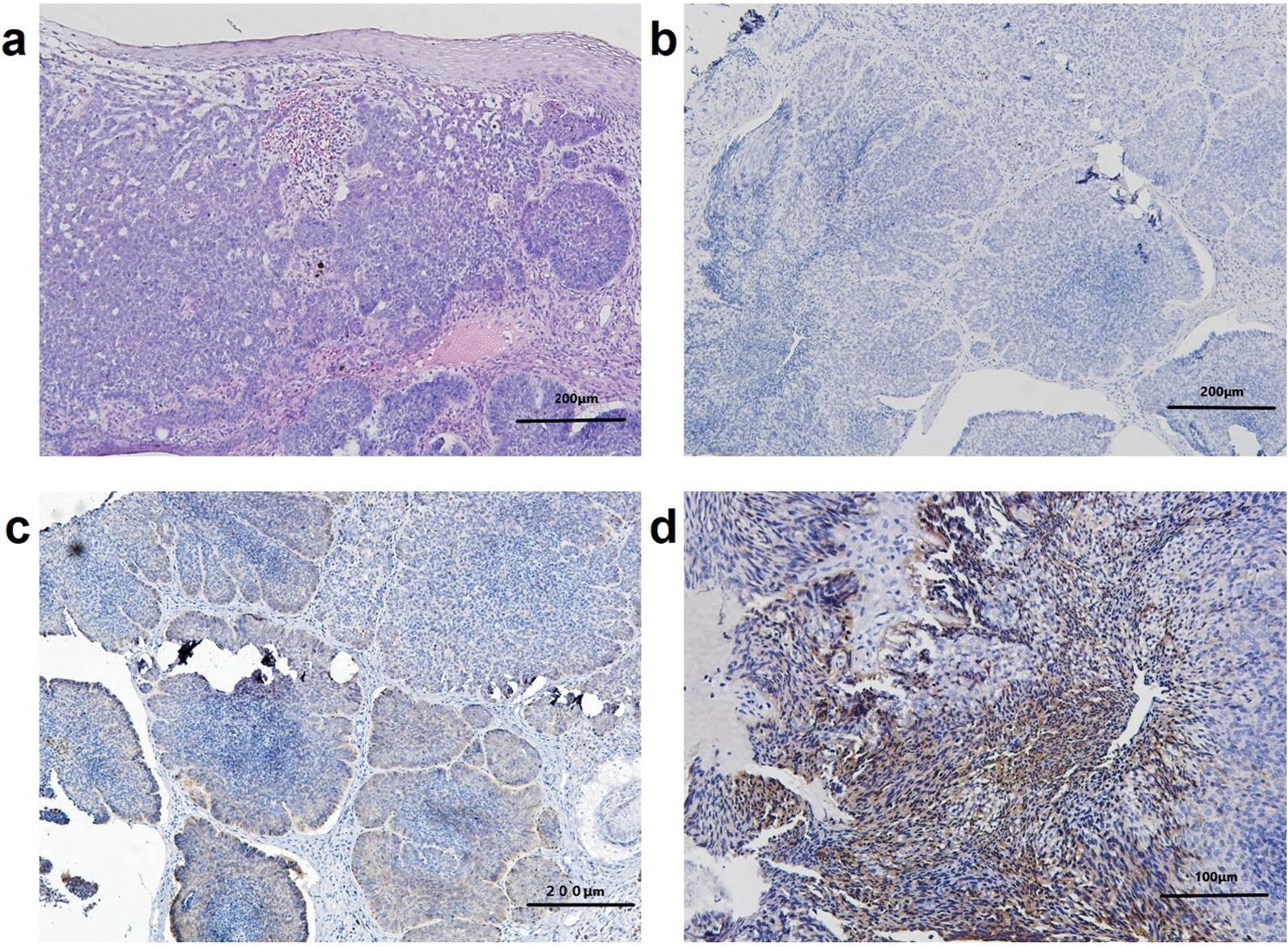
Histopathologic examination of biopsy specimen showed compact basaloid cell nests with peripheral palisading that extend into the dermis (**a**, HE × 100). Except for CK10 (**b**, SP × 100), Bcl-2 and BerEp4 immunoreactivity were observed (**c** and **d**, SP × 100 and 200, respectively)

In this family, the earliest onset of the disease was at 16 years old, and the average onset age was 19 years. The proband (II-6) complained of multiple hyperpigmented nodules on her body (Fig. 2a). She had ocular hypertelorism, but no obvious palmar and/or plantar pits. Cranial CT and upper-limb X-ray showed ectopic calcifications of the falx cerebri and abnormalities in bone density, respectively (Fig. 2b and c). Histopathologic examination of biopsy specimen from her right face revealed compact basaloid cell nests with peripheral palisading that extend into the dermis (Fig. 3a). Except for CK10 (Fig. 3b), Bcl-2 and BerEp4 immunoreactivity were observed (Fig. 3c and d). There were three other family members affected by this disease. Hence, the diagnosis of GS was made based on three major and two minor criteria, established in 2011 [10]. Among them, the patient (II-2) died of cachexia resulting from aggressive BCCs on her neck four months after the diagnosis. Similarly, it was told that the patient (I-1) died of giant BCCs on his nose in 2010.

### Germline and somatic mutations in the PTCH1 tumor suppressor gene

In this study, the paternal germline mutation c.1341dupA (p.L448Tfs*49) in *PTCH1* cosegregated with multiple BCCs (Fig. 1b), which was identified by WES and confirmed by Sanger sequencing. To our knowledge, this insertion mutation has been listed on HGMD^®^ and molecular genetics of Kitasato University School of Medicine website. But no clinical data are available from those patients. Moreover, we analyzed WES data from 6 paired BCC/ANCT and their matched blood samples. As Table 1 indicated, different somatic *PTCH1* mutations were found in four of the six paired BCC samples. Those somatic mutations were present on the wild-type maternal allele, which was confirmed by subcloning of genomic DNA and Sanger sequencing. Moreover, additional driver mutations in *ERBB2* and *PPP6C* were found in two of the six BCCs (Additional file 1). Loss of heterozygosity of *PPP6C* and *PTCH1* were observed in two and three of the six BCCs, respectively (Additional file 2). These results showed the two-hit mutational inactivation of *PTCH1* and the genomic heterogeneity in BCCs. These results revealed the genomic heterogeneity in BCCs, as supported by previous findings [11].

**Table 1:** Somatic *PTCH1* mutations found in four of the six paired BCC samples

No. in the pedigree chart	Sex	Age (years)	Location of skin biopsies	Paired skin Samples	No. of somatic mutation	TMB (mutations/MB)	Somatic mutations of *PTCH1* in BCC	Reads in BCC, no.		Reads in ANCT, no.	
								Ref.	Non-ref.	Ref.	Non-ref.
II. 2	Female	39	Left side of the neck	II.2-1_BCC/ ANCT	346	10.81	c.391G > T (p.Glu131*)	33	10	66	0
			Abdomen	II.2-2_BCC/ANCT	39	1.22	c.3271G > C (p.Gly1091Arg)	196	13	148	0
II. 4	Female	36	Behind the right ear	II.4-1_BCC/ANCT	386	12.06	Negative				
			Back of the neck	II.4-2_BCC/ANCT	200	6.25	c.1396C > T (p.Gln466*)	139	28	222	0
II. 6	Female	29	Right side of the nose	II.6-1_BCC/ANCT	567	17.71	Negative				
			Left side of the nose	II.6-2_BCC/ANCT	989	30.9	c.1130dupA (p.Tyr379Valfs*58)	142	11	159	0

*non-ref.* non-reference; *ref.* reference; *TMB* tumor mutation burden; *MB* mega base

### Distinct mutational signatures of the BCCs from three sisters

**Fig. 4:**
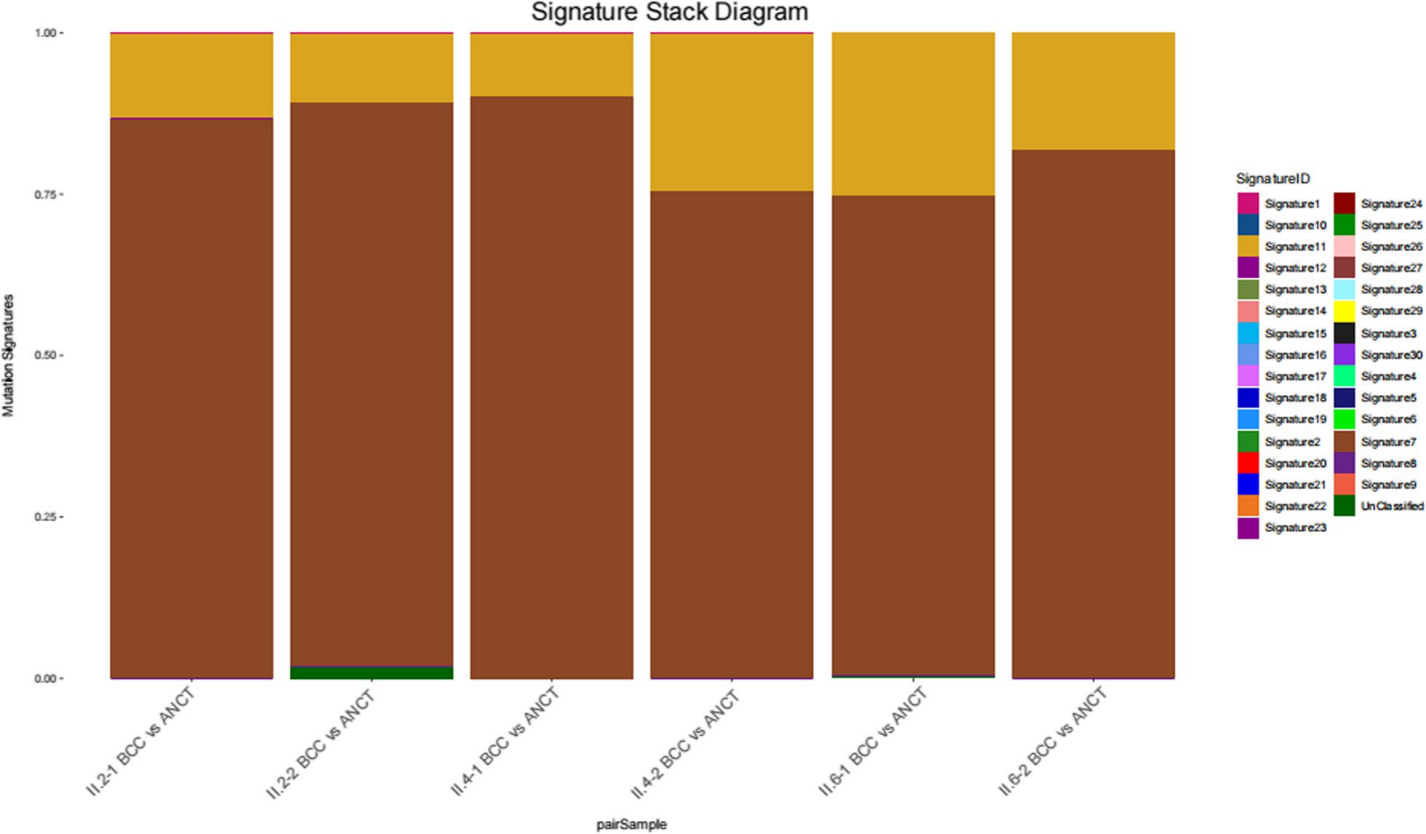
Two distinct mutational signatures were present in six pairs BCC and ANCT, which are Signature7 (brown) and Signature11 (yellow)

The mutational 0signature analysis was performed on the exomes of six BCC-ANCT pairs, inferring DNA damage and repair processes during the evolution of cancer. As indicated in Fig. 4, Signature 7 and 11 were distinctly detected in BCCs with either one or two *PTCH1* mutations using the deconstructSigs R package. Of note, Signature 7 in a brown color predominated over Signature 11 in a yellow color. It is known that Signature 7 represents ultraviolet (UV) light related patterns of mutations, which show C > T transitions at dipyrimidines and CC > TT double nucleotide substitutions [12]. Whereas Signature 11 is statistically associated with the C > T substitutions pattern of agents treatment [13]. Both Signature 7 and 11 are non-clock-like mutational signature patterns, which are associated with better prognosis [14]. It might explain why GS has little effect on the life expectancy of patients.

### Loss-of-function and over-expression of PTCH1 leading to activation of the Hh pathway in BCCs

**Fig. 5:**
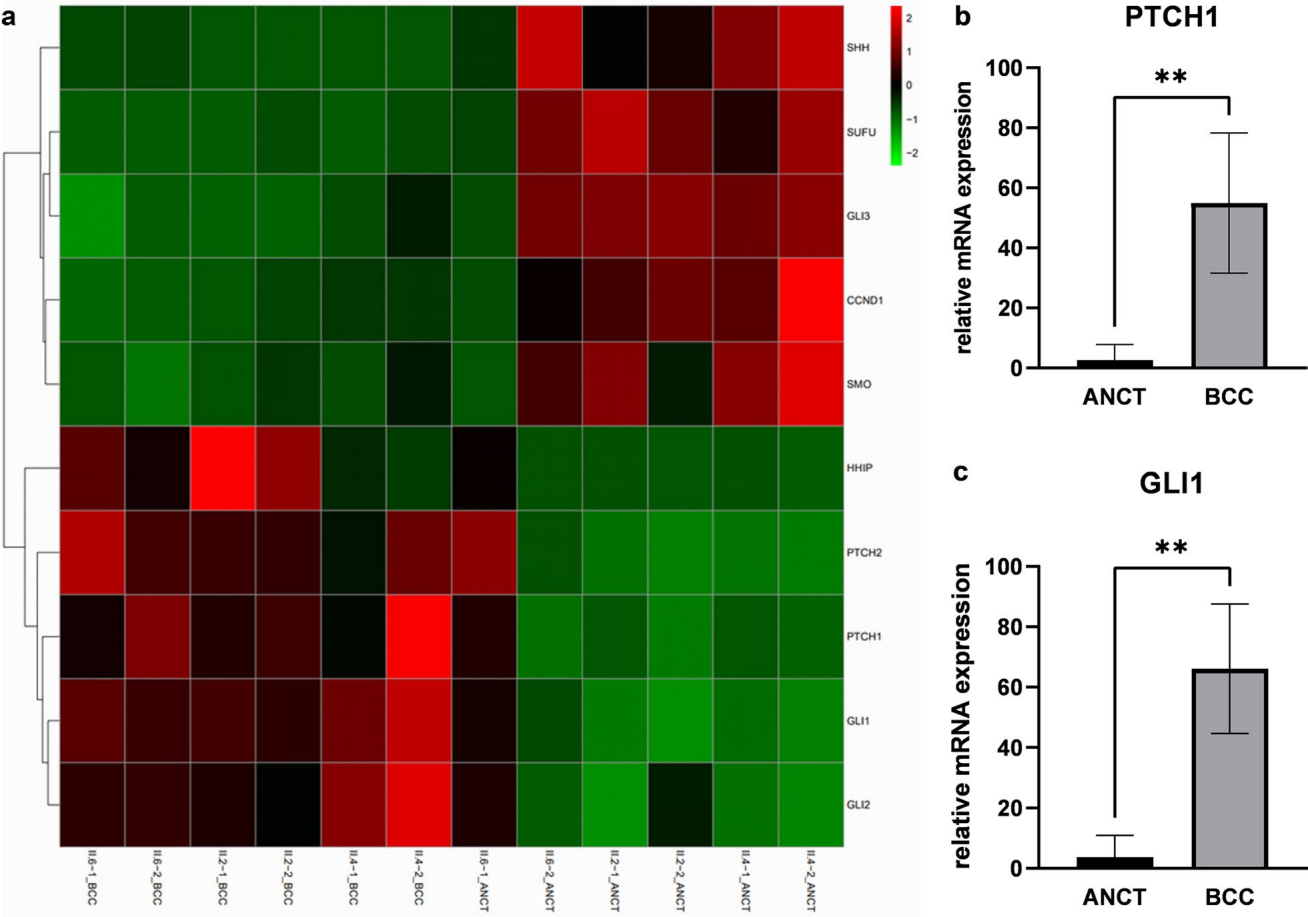
**a** Hierarchical clustering and heatmap representation of 10 differentially expressed Hedgehog signaling pathway genes. Clustering was performed on six pairs of BCC and ANCT from three sisters with GS. Expression levels are colored with a red-to-green gradient, where red and green represented up-regulation and down-regulation, respectively. The expression levels of *PTCH1* **b** and *GLI1* **c** were significantly up-regulated in six pairs of BCC versus ANCT, confirmed by real-time qPCR. **p value < 0.01 (two-tailed paired-sample t-tests; n = 12)

Transcriptional analysis by RNA-seq showed up-regulation of the Hh pathway genes in BCCs (Fig. 5a). In particular, our finding showed that *PTCH1* and *PTCH2* shared similar expression patterns with *GLI1* and *GLI2*. We quantified the expression of *PTCH1* and its target *GLI1* genes in six pairwise samples to confirm the results obtained by RNA-seq. The results indicated that the transcript levels of *PTCH1* (Fig. 5b) and *GLI1* (Fig. 5c) were relatively higher in BCCs compared with ANCT. The presence of inactivating mutations in *PTCH1* promote autocrine activation of Hh signaling and genesis of BCCs. It is reported that the binding of Hh ligands to up-regulated *PTCH1* receptors can activate smoothened (*SMO*) and lead to the overexpression of down-stream transcription factors. Moreover, transcription factor *GLI2* directly activates *GLI1* and forms a positive feedback loop to promote BCCs [15, 16].

## Discussion

It is known that GS is not a life-threatening illness and has a near-normal life expectancy [2]. Except that complication was given as the underlying cause of death, around 10% (3/32) of deaths has been reported due to invasive lesions and intracranial involvement [17, 18]. However, in this three-generation GS family, one male (I-1) and one female (II-2) died as a direct result of aggressive BCCs, whose ages were 54 and 39 years respectively. There were three main reasons for their untimely death, including driver gene mutations, exposure to intense UV radiation and the poor economic conditions.

To our knowledge, the activation of Hh pathway represents the initial tumorigenic event in BCCs. In this study, the *PTCH1* c.1341dupA (p.L448Tfs*49) mutation was identified in this GS family. Besides this germline mutation, different somatic *PTCH1* mutations were found in four of the six paired BCC samples. These findings provided the evidence of two-hit mutational inactivation of *PTCH1* and the genomic heterogeneity of BCCs, which supported the Knudson’s two-hit tumor progression hypothesis [19]. It is generally accepted that *PTCH1* is one of the most common driver genes in BCCs. The concept of “driver” and “passenger” genes in cancer was proposed by Stratton et al. [20]. In contrast to the passenger gene, the driver gene is critical for oncogenesis. The driver and passenger gene mutations might be regarded as “mountain” and “hill” [21], respectively. The driver mutations are defined as making important contributions to cancer development, as compared to the passenger mutations. Previous studies have suggested the driver pivotal role of *PTCH1*, *TP53*, and *SMO* in BCCs development [22]. Moreover, *PPP6C* and *ERBB2* are reported as additional driver genes in BCCs [11], which were also found in two of the six BCC samples from three sisters. The major limitation of this study was that we didn’t perform additional confirmation of these driver genes due to a limited budget and time.

The three-generation family with GS lives in a rural area of Hainan province, located in the southernmost part of China. This is the reason that they are exposed to intense UV radiation for a long time. It is believed that UV radiation plays a crucial role in the onset of skin carcinogenesis through DNA damage and immune suppression. Of note, UVB is directly absorbed by DNA and induce UV-signature DNA damages [23]. In this study, the results of WES showed that Signature 7 and 11 were mainly present with similarities in six pairs BCC versus ANCT, making it possible to decipher their mutational patterns. It is discovered that Signature 7 shows a higher prevalence of C > T mutations [13], attributed to pyrimidine dimers caused by UV exposure. In addition, Signature 11 exhibits a mutational pattern resembling that of alkylating agent treatment, associated with mutations occurred on guanine [24]. However, it is said that this family never underwent chemical treatment, and possible reasons need to be further explored. As mentioned above, both Signature 7 and 11 belong to non-clock-like mutational patterns, which means those mutational processes may occurred in an episodic manner, generating sudden mutations in a short time, rather than at a steady rate [14]. By comparison, clock-like signature is associated with worse prognosis, tumor progression and immune resistance to immune checkpoint inhibitor therapy [25]. Moreover, those non-clock-like mutational signatures might be consistent with the near-normal life expectancy of GS patients.

Notably, poor economic conditions of this family were the primary reason for the delay in seeking medical treatment. In recent years, our government provided special funding to support medical examinations in rural areas. The two patients who died might have survived longer if they went to hospital and received surgical treatment in time.

## Conclusions

Taken together, this study provided clinical information and mutation analyses of *PTCH1* in a three-generation family with GS, which has been listed on HGMD^®^. The main reasons for untimely deaths of GS patients were driver gene mutations, exposure to intense UV radiation and the poor economic conditions. Although the genetic background can’t be changed, it has been well established that restricting exposure to UV light is mandatory for GS patients.

## Supplementary Information

**Additional file 1:** Somatic single nucleotide variant (SNV) sites in whole exome sequencing of matched BCC and ANCT.

**Additional file 2:** Somatic loss of heterozygosity (LOH) in whole exome sequencing of matched BCC and ANCT.

## Data Availability

The datasets generated during the current study are available in the [Mendeley Data] repository, Peng, Kexin; Zhang, Zhenghua (2022), “Distinct Non-Clock-Like Signatures of the Basal Cell Carcinomas from Three Sisters with a Lethal Gorlin-Goltz Syndrome”, Mendeley Data, V3, https://doi.org/10.17632/5s4pk4rj32.3.

## Abbreviations

GS: Gorlin-Goltz syndrome
BCCs: Basal cell carcinomas
Hh: Hedgehog
WES: Whole exome sequencing
ANCT: Adjacent non-cancerous tissues
UV: Ultraviolet
NBCCS: Nevoid basal cell carcinoma syndrome
PTCH1: Patched 1
PTCH2: Patched 2
SUFU: Suppressor of fused
GATK: Genome Analysis Toolkit
SNVs: Single nucleotide variants
GVCF: Genomic variant call format
VQSR: Variant quality score recalibration
VEP: Variant effect predictor
MAF: Mutation annotation format
